# Strengthening national salt reduction strategies using multiple methods process evaluations: case studies from Malaysia and Mongolia

**DOI:** 10.1017/S1368980023002781

**Published:** 2024-02-12

**Authors:** Briar L McKenzie, Feisul Idzwan Mustapha, Bat-Erdene Battumur, Enkhtungalag Batsaikhan, Arunah Chandran, Viola Michael, Jacqui Webster, Kathy Trieu

**Affiliations:** 1 The George Institute for Global Health, UNSW, Level 18, International Towers 3, 300 Barangaroo Ave, Barangaroo, NSW 2000, Australia; 2 Ministry of Health Malaysia, Putrajaya, Wilayah Persekutuan, Malaysia; 3 Department of Public Health, Ministry of Health, Ulaanbaatar, Mongolia; 4 Department of Nutrition Research of the National Center for Public Health, Ulaanbaatar, Mongolia

**Keywords:** Salt reduction, Process evaluation, Mongolia, Malaysia

## Abstract

**Objective:**

To understand the extent to which national salt reduction strategies in Malaysia and Mongolia were implemented and achieving their intended outcomes.

**Design:**

Multiple methods process evaluations conducted at the mid-point of strategy implementation, guided by theoretical frameworks.

**Setting:**

Malaysia (2018–2019) and Mongolia (2020–2021).

**Participants:**

Desk-based reviews of related documents, interviews with key stakeholders (*n* 12 Malaysia, *n* 10 Mongolia), focus group discussions with health professionals in Malaysia (*n* 43) and health provider surveys in Mongolia (*n* 12).

**Results:**

Both countries generated high-quality local evidence about salt intake and levels in foods and culturally specific education resources. In Malaysia, education and reformulation activities were delivered with moderate dose (quantity) but reach among the population was low. Within 5 years, Mongolia implemented education among schools, health professionals and food producers on salt reduction with high reach, but with moderate dose (quantity) and reach among the general population. Both countries faced challenges in implementing legislative interventions (mandatory salt labelling and salt limits in packaged foods) and both could improve the scaling up of their reformulation and education activities.

**Conclusions:**

In the first half of Malaysia’s and Mongolia’s strategies, both countries generated necessary evidence and education materials, mobilised health professionals to deliver salt reduction education and achieved small-scale reformulation in foods. Both subsequently should focus on implementing regulatory policies and achieving population-wide reach and impact. Process evaluations of existing salt reduction strategies can help strengthen intervention delivery, aiding achievement of WHO’s 30 % reduction in salt intake by 2025 target.

There is strong and consistent evidence that excess Na intake raises blood pressure which increases CVD risk^(1,2)^. It was estimated that in 2017, excess Na intake (mostly in the form of salt) was responsible for 3 million non-communicable disease deaths worldwide^(3,4)^. Systematic reviews of randomised controlled trials consistently show that reducing salt intake can lower blood pressure in all population groups^(1,2)^. Given the extensive evidence base, the WHO has called for Member States around the world to reduce mean population salt intake by 30 % by 2025, as one of nine global targets to reduce premature death from non-communicable diseases by 25 %^(5)^.

To assist countries in prioritising interventions, the WHO recommended four ‘best buy’ interventions to lower salt intake based on evidence that they were effective, cost effective, feasible and low cost^(5)^. These included (1) reformulate food products to contain less Na by setting targets, (2) enable lower Na options to be provided in public institutions, (3) introduce behaviour change and mass media communication and (4) implement front-of-pack nutrition labelling^(5)^. In 2019, despite ninety-six countries implementing one or more of the ‘best buy’ salt reduction interventions, only three countries have demonstrated a substantial (>2 g/d) decrease in mean salt intake, whilst fourteen countries demonstrated a moderate to slight decrease (<2 g/d)^(6)^. More evaluations and a better understanding of how salt reduction strategies are implemented and/or under what context the interventions are effective could help more countries achieve meaningful reductions in population salt intake.

One reason for the lack of evaluations of salt reduction strategies is the cost, complexity and resource-intensive task of accurately measuring population salt intake through the gold standard method of 24 h urine collection^(7)^. While there are other methods such as estimating salt intake from spot urine samples and dietary assessment methods, their accuracy remains suboptimal compared to 24 h urine collection^(7,8)^. While not an alternative to measuring salt intake, process evaluations can be conducted more easily and frequently to assess the extent to which the intervention is being delivered as planned and achieving its intended effects. Process evaluations can also identify the contextual barriers hindering implementation and salt reduction that need to be overcome and enablers to leverage^(9)^. While process evaluations of salt reduction interventions have been conducted in the past^(10,11)^, they were conducted retrospectively, after the interventions were completed.

Thus, our study illustrates how process evaluations conducted during implementation can be used to develop context-specific recommendations for strengthening salt reduction strategies, with Malaysia and Mongolia as case studies. Both are classified as a low- to middle-income country (Mongolia classified as a lower middle-income country, Malaysia classified as an upper middle-income country^(12,13)^). In both countries, salt intake exceeded the recommended limit of 5 g/d, estimated at 8·7 g/d in Malaysia^(14,15)^ and 11·1 g/d in Mongolia^(16)^. In Malaysia, the main dietary sources were discretionary salt and salty sauces (for example, soya and oyster sauce)^(14,15)^, whereas in Mongolia, salted tea, processed and smoked meat products, pickled vegetables and processed foods were the main dietary contributors^(16)^. As such, both had government-led National Salt Reduction Strategies (from now on referred to as the ‘Strategies’ or ‘Strategy’) spanning 5 years in Malaysia and 10 years in Mongolia. Both countries recognised that interim process evaluations around the midpoint were needed to understand how the strategies were tracking and how they could be enhanced to aid the achievement of the salt reduction target.

## Methods

### Theoretical framework

We followed guidance on process evaluations of complex interventions from the UK Medical Research Council^(9)^. The Guidance focuses on examining three related aspects, *implementation, mechanism* and *context*, to understand progress to date, if activities are causing expected outputs, areas that need strengthening and facilitators to leverage in future implementation (Table 1).

**Table 1:** Process evaluation dimensions and data sources

Evaluation dimensions	Definition	**Data sources** *(primary source bolded)*
Reach	The number or proportion of the intended target audience that is exposed to the intervention	**Desk review of documents** and semi-structured interviews
Dose	Dose delivered: the quantity or amount of intervention delivered or provided. Dose received: the extent to which participants engage with the intervention.	**Desk review of documents** and semi-structured interviews.
Adoption	The number or proportion of organizations or implementers who engage in the intervention or adopt the intervention	**Semi-structured interviews** and desk review of documents.
Fidelity	The integrity and quality of the intervention delivered compared to what was intended	**Semi-structured interviews** and desk review of documents.
Mechanism of impact	The pathway of impact from the delivery of the intervention to outcome change.	**Semi-structured interviews** and desk review of documents
Contextual influences of implementation	Factors external to the intervention that impede intervention delivery and the factors that facilitate intervention delivery.	**Semi-structured interviews**, desk review of documents, focus group discussions or questionnaires^*^
Contextual influences of outcomes	Factors external to the intervention that act as a barrier or facilitator of the intervention effect.	**Focus group discussions or questionnaires** * and desk review of documents.

* Focus group discussion were used in Malaysia, and questionnaires were used in Mongolia.

First, the *implementation* of each intervention component was thoroughly examined through the four dimensions; reach, fidelity, dose and adoption as defined by Steckler and Linnan’s framework^(17)^.

Second, the *mechanism of impact* (i.e. how the interventions caused change in salt intake) was examined in terms of whether the intervention produced the assumed change or unexpected consequences and how each intervention component interacted with one another to produce the intended outcome. To illustrate this, we developed logic models specific to each country’s strategy (see online supplementary material, Supplementary Figures 1 and 2), detailing the inputs, activities, outputs and planned outcomes for each intervention within the strategies.

Third, two types of *contextual influences* were examined; factors (barriers and facilitators) that influenced (1) the delivery of the intervention and (2) the intervention outcome: whether reduced salt consumption was achieved, and the extent achieved, in each country.

### Data collection

The interim evaluations were conducted between November 2018 and April 2019 in Malaysia and from March 2020 till March 2021 in Mongolia. A multiple methods approach comprising three main data sources were used to collect information about the *implementation, mechanism of impact* and the *contextual influences* of intervention delivery and salt reduction (Table 1). The data sources included:

A desk-based document review of activity logs and internal reports provided by the program implementers and other involved organisations and publicly available information.

Semi-structured interviews (see online supplementary material, Supplementary Tables 1 and 2) with key informants directly or indirectly involved in the implementation, or impacted by, the salt reduction strategy. Interview guides were designed to gain a richer understanding of the extent of implementation particularly the intervention fidelity, hypothesised mechanism of impact and barriers and facilitators of intervention delivery and salt reduction in each country.

For Malaysia, focus group discussions with a range of stakeholders (detailed in Table 2) were conducted using the nominal group technique^(18)^ to identify and prioritise the barriers or facilitators of lowering salt intake among adults. For Mongolia, due to COVID19 restrictions, focus group discussions were not possible, and instead a questionnaire on barriers and facilitators of lowering salt consumption in Mongolia were sent to health department workers and community health workers (see online supplementary material, Supplementary Table 3).

**Table 2:** Data collection steps and differences in data collection between Malaysia and Mongolia

Step		Deviations/country-specific data collection
1.	Desk-based document review of activity logs and internal reports provided by the program implementers and other involved organisations and publicly available information.	*Same methods for Malaysia and Mongolia.*
2.	Semi-structured interviews with key informants directly or indirectly involved in the implementation, or impacted by, the salt reduction strategy.	Malaysia – twelve purposively selected key informants were interviewed in person, including representatives from different divisions of the Ministry of Health in Malaysia, local Universities, state health department community health professionals, non-governmental organizations (NGO) and food industry.
		Mongolia – ten purposively selected key informants were interviewed by zoom, seven government officials and three NGO or industry association representatives.
3.	Discussions with, or survey of, perspectives on barriers and facilitators to salt reduction strategy implementation.	Malaysia – Three focus groups discussions conducted in person with forty-three participants. Participants represented state-health department representatives from all states in Malaysia, representatives from the different divisions of Ministry of Health, NGOs, Universities and the food industry were also conducted. The nominal group technique process was used to identify and prioritise the current barriers or facilitators of lowering salt intake among adults.
		Mongolia – Questionnaires translated into Mongolian were sent via email to provincial health professionals. The questionnaire listed a range of possible barriers and facilitators of lowering population salt intake along with an open-ended question for individuals to list any other perceived barriers or facilitators, not already listed. Individuals were asked to rank the barriers and then to rank the facilitators from the most important to the least important. Twelve questionnaires were completed and returned.

Further information on country specific data collection is shown in Table 2.

### Data analysis

For each country, data from the desk-based document review relating to the process evaluation dimensions (reach, dose, adoption and fidelity) were extracted and organised under the three main interventions and their related activities in a spreadsheet. Semi-structured interview responses were transcribed by an independent company, and any personally identifiable information was removed. Investigators coded the responses in NVivo (version 12; QSR International, Doncaster). A deductive and inductive approach was used to code relevant interview responses relating to the reach, dose, adoption, fidelity, barriers of implementation and facilitators of implementation for each activity under the three interventions. Where possible, data from the interview responses and documents identified in the desk review were used to validate one another.

For Malaysia, priority barriers and facilitators to the strategy and to consuming less salt generally were determined by summing individuals’ rankings obtained from the nominal group technique process. Whereas for Mongolia, questionnaire responses on the barriers and facilitators of reducing salt consumption were collated in a spreadsheet and similar factors were grouped together to identify the most commonly reported barriers and facilitators of salt reduction in Mongolia. These barriers and facilitators were also categorised according to Story et al’s ecological framework^(19)^, to understand the levels of influence on what people eat (i.e. individual factors, social environments, physical environments and macro-level environments). The findings were used to examine whether the current interventions were addressing the main barriers of salt reduction in each country, and if not, what new activities were needed; what facilitators to leverage and to help prioritise salt reduction activities.

### Formulation of recommendations

Three main considerations were used to formulate the priority recommendations for strengthening the salt reduction strategy. First, activities that would have a large impact on achieving the targeted outcomes were determined by examining the implementation progress, outputs and planned mechanism of impact depicted in the logic model. Second, activities where there were feasible solutions to overcome the implementation challenges were prioritised. Third, recommendations that address the top barriers of lowering salt intake in the country were prioritised. The recommendations were discussed with program implementers in Malaysia and Mongolia to ensure they were feasible to adopt, that there was a clear rationale for the recommendations, and to get their buy-in.

## Results

### Malaysia

Malaysia’s salt reduction strategy *‘Salt Reduction Strategy to Prevent and Control NCD for Malaysia (2015–2020)’* is led by the Ministry of Health and aimed to reduce population salt intake by 15 % by 2020. This strategy comprised of three focus areas: (1) Monitoring population salt intake and Na in foods (***monitoring***), (2) generating awareness about the need to reduce salt intake (***awareness***) and (3) lowering the Na content of food products through industry engagement, reformulation and labelling schemes (***products***) (see online supplementary material, Supplementary Figure 1).

#### Extent of implementation

Overall, during 3 years of implementation most of the strategy components had been implemented with high quality/fidelity; however, the dose of the intervention delivered and adopted was moderate, and the reach of the interventions among the Malaysian population was low. Overall, the awareness raising activities were the most-well implemented, followed by monitoring activities and, least of all, activities related to food products.

The extent of implementation differed by strategy component (detailed in Table 3):

**Table 3:**
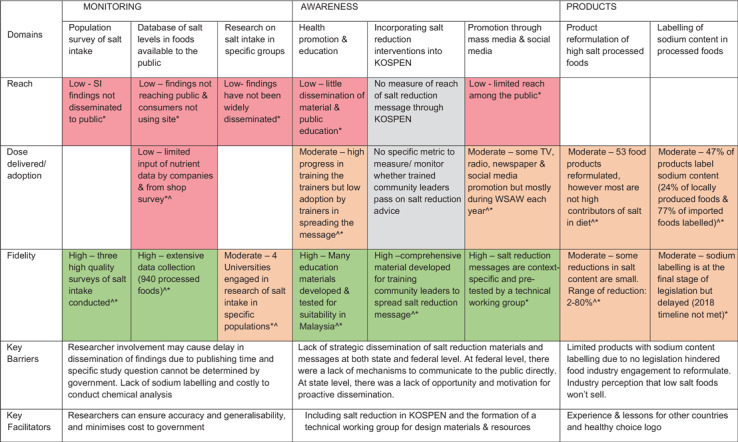
Summary of the implementation of the salt reduction strategy by dimensions, Malaysia

KOSPEN stands for ‘Komuniti Sihat Perkasa Negara’ translates to *Strong National Healthy Community.* WSAW stands for World Salt Awareness Week.

*Based on qualitative data from semi-structured interviews or focus groups.

†Based on data from routinely collected data or publicly available data. Domains are scored low if there is limited implementation, moderate refers to some activity but more is needed and high refers to adequate level of effort.

##### 1. Monitoring

Most interventions related to *monitoring* population salt intake and Na content in processed foods had been implemented with high fidelity and quality. Two surveys used 24-h urine collection to estimate salt intake, the gold standard method for salt intake estimation. Surveys of packaged foods were also conducted, assessing whether Na was labelled on back-of pack nutrition labels and the level of salt content in foods. While high-quality surveys were conducted, stakeholders reported there was limited dissemination of survey findings to the general population (for awareness raising) and food companies (to encourage reformulation) meaning there was low reach (Table 3). Stakeholders explained that the dissemination of survey findings were delayed because of the need to wait for the research to be published.

##### 2. Awareness

Like the monitoring activities, the *awareness* raising initiatives have achieved high fidelity and quality, moderate dose delivered and low reach. A comprehensive range of context-specific, high-quality salt reduction resources (such as infographics, videos, recipe books, a manual on how to use education materials and salt reduction messages) had been developed for health education, mass media activities, as well as an existing community intervention (Table 3). However, there was moderate use of such education materials and low dissemination and therefore low reach among the public. Most high reaching education activities were only conducted once a year during World Salt Awareness Week. Stakeholders suggested this is due to a lack of affordable mechanisms to deliver messages to the public directly, and thus mass media activities were limited to once a year. Additionally, while several trainers (state level health care professionals such as community nutritionists, medical doctors and dietitians) received training on how to use the salt reduction educational materials, there was limited dissemination to their fellow trainers and the public compared to what was intended. Interviewees explained this was because there was a lack of motivation and opportunity for proactive dissemination.

##### 3. Products

The *products* intervention consisted of two main activities, namely, encouraging food companies to reformulate high-salt processed foods and labelling (or declaration) of Na content on processed foods. These activities had achieved moderate fidelity and adoption. At the time of the interim evaluation, fifty-three food products (e.g. instant noodles, cakes, sauces, biscuits, snacks, frozen meats, dressing and tea and 3-in-1 drinks) had been reformulated; however, the extent of reformulation varied, from as little as 2 % to an 80 % reduction in Na content from previous formulation. Further, only some reformulated products were high Na contributors or market leaders, meaning the impact of reformulation on Malaysian’s Na intake may be limited. Legislation on mandatory labelling of Na content on back-of-pack nutrition labels was due to be endorsed by 2018; however, at the time of evaluation, stakeholders reported it had been delayed due to competing priorities with the need to incorporate sugar labelling into the legislation. There had also been efforts to encourage food industry to voluntarily label Na content on packaged foods by making it a pre-requisite for the application to use of the ‘healthy choice logo’; however, only 47 % of products voluntarily declared the Na content (Table 3). Program implementers reflected that the lack of complete Na content labelling across food products hindered activities to engage food companies in reformulation as it was difficult to know which high-salt products or companies to target and targeting reformulation among products that voluntarily declared Na levels could discourage labelling.

### Mongolia

Mongolia has a 10-year national salt reduction strategy (2015–2025), led by the National Centre for Public Health, within the Ministry of Health. This strategy is composed of three key objectives: (1) *to create an enabling legal environment for promoting the production, importation, marketing and service of lower salt foods* (**legal environments**); (2) *to improve partnerships between government and private sector to reduce salt content of foods and by increasing controls on production, service, marketing, importation and consumption of food* (**private sector**) and (3) *to create an enabling environment which supports people to develop habits of optimal salt intake and make healthy food choices* (**support people/consumers**). Within each objective there are several specific activities that together aim to reduce mean adult salt consumption by 30 % (to 7·8 g/d) by 2025 (see online supplementary material, Supplementary Figure 2).

#### Extent of implementation

Overall, during the first 5 years (2015–20), Mongolia’s salt reduction strategy has been implemented with mostly high fidelity, reach and dose or adoption (Table 4), with consensus among all interviewees that the strategy has been implemented well. Of the three intervention objectives, intervention objective 2, to engage the private sector, had been particularly well-implemented, followed by objective 3, to support people to reduce their salt consumption, and objective 1, to create legislation to support salt reduction.

**Table 4:**
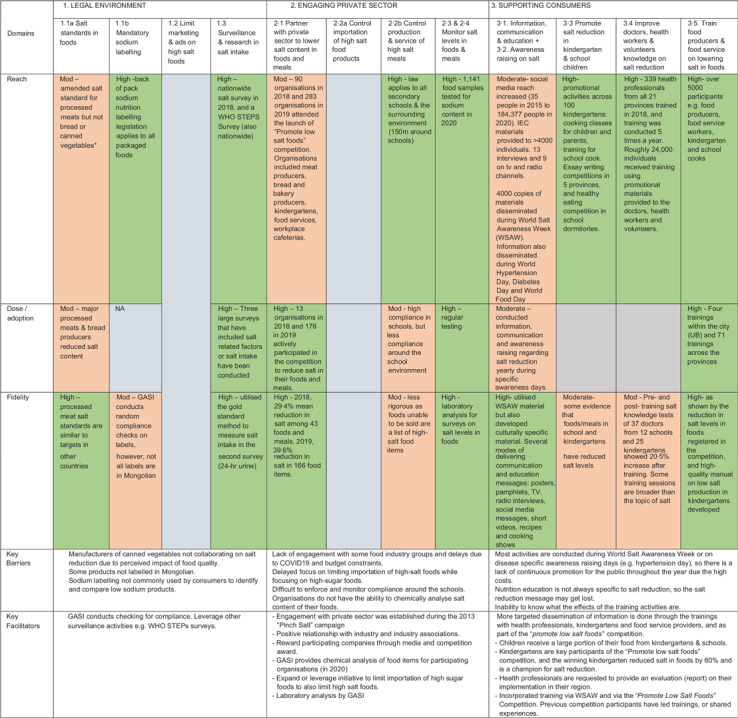
Summary of the implementation of the salt reduction strategy by dimensions, Mongolia

GASI stands for ‘The Generalised Agency for Specialised Inspection’. IEC stands for ‘Information, communication and education’. WSAW stands for ‘World Salt Awareness Week’.

The extent of implementation differed by strategy component (Table 4):

##### 1. Legal environment

The key activities under objective 1 ‘legal environment’ consisted of: amending salt content standards for foods and technical regulation to mandate Na labelling; limit marketing and advertising of high-salt foods and conduct surveillance and research on salt intake. For this objective, it was found that activities have had a moderate to high reach, dose, adoption and fidelity (Table 4). The most strongly implemented activity was the surveillance and research on salt intake – with three surveys using high-quality methods conducted in the past years (Table 4). Salt standards have had moderate adoption with salt standards tightened for processed meats, no formal change in standards but reduced Na content in breads and no changes in canned vegetables. Stakeholders reported that canned vegetable producers perceived reducing the salt content would negatively impact the quality of products. Legislative activities to restrict the marketing and advertising of high-salt foods had yet to be established.

##### 2. Engaging private sector

The key activities under objective 2 ‘engaging private sector’ consisted of: engaging private sector to increase the production, marketing and service of lower salt foods and meals; enforcing governmental controls on the importation, production, service and marketing of high-salt foods and meals; implementing internal monitoring of the salt content of processed foods. There had been substantial progress on activities related to *engaging private sector*, with reach, dose, adoption and fidelity of activity implementation classified as moderate to high (Table 4). A competition called ‘Promote Low Salt Foods’ achieved high adoption and fidelity as it encouraged 176 food producers, food caterers, restaurants and food service providers to lower salt levels in their foods and meals; however, the competition could have had a wider reach if it was publicised to others beyond the competing food service providers (Table 4). Efforts to control the importation of high-salt products were in the early stages, with a proposal for this submitted to government; however, stakeholders described that the focus is currently on high-sugar foods rather than high-salt foods. More progress had been made towards controlling the production and service of high-salt meals, as mandatory regulations were put in place in 2020 to limit high salt (and unhealthy foods more broadly), within and around secondary schools. The reach of this activity has been high, as the regulation applies to all secondary schools, and there is evidence that compliance within schools is high; however, stakeholders perceived that compliance was less around schools, as it was difficult to monitor or enforce in the area around schools. Finally, the monitoring of salt levels of meals and food products was ranked as high, with systems in place for the regular testing of food samples (Table 4).

##### 3. Support people to reduce salt intake

The key activities under objective 3 ‘supporting people to reduce salt intake’, consisted of: information communication and education activities, promotion of salt reduction (with a focus on kindergarten and school aged children), improving health care professionals’ knowledge on salt reduction and training of food producers and food services on lowering salt in food. Most of the communication and education campaigns to improve public awareness were delivered on specific ‘awareness’ days (e.g. World Salt Awareness Week) that usually occurred once a year, meaning the dose delivered was moderate. The campaigns achieved moderate reach through dissemination of materials and some television and radio interviews. However, the fidelity was high as numerous high-quality, culturally specific salt reduction resources were developed for a range of different modes of communication. The reach of the salt reduction promotion activities among kindergarten and school children was high as over 100 kindergartens and schools participated in cooking demonstrations and writing competitions about salt reduction (Table 4). However, there was only some evidence that salt levels of foods/meals in kindergartens and schools had been reduced and no evidence of an impact on salt-related knowledge or behaviours of participants. Similarly, the reach of the training of health workers and volunteers to improve their knowledge around salt reduction was high with over 300 health professionals participated, across all twenty-one provinces in Mongolia trained; however, there were moderate improvements in salt-related knowledge following training, and it was unclear the extent to which health care professionals passed on salt reduction information to the community. The final activity, on regular training for food producers and food service personnel, was the most strongly implemented with evidence of high reach, dose and fidelity. Over 5000 people had participated in a training, and there was evidence of multiple training sessions held across provinces in Mongolia. This activity links with the ‘Produce low salt foods’ competition (see *2. Engaging private sector*) with evidence that food producers and food services had reduced the salt content of food products and meals by a significant amount (Table 4).

## Barriers and facilitators affecting salt reduction

**Fig. 1:**
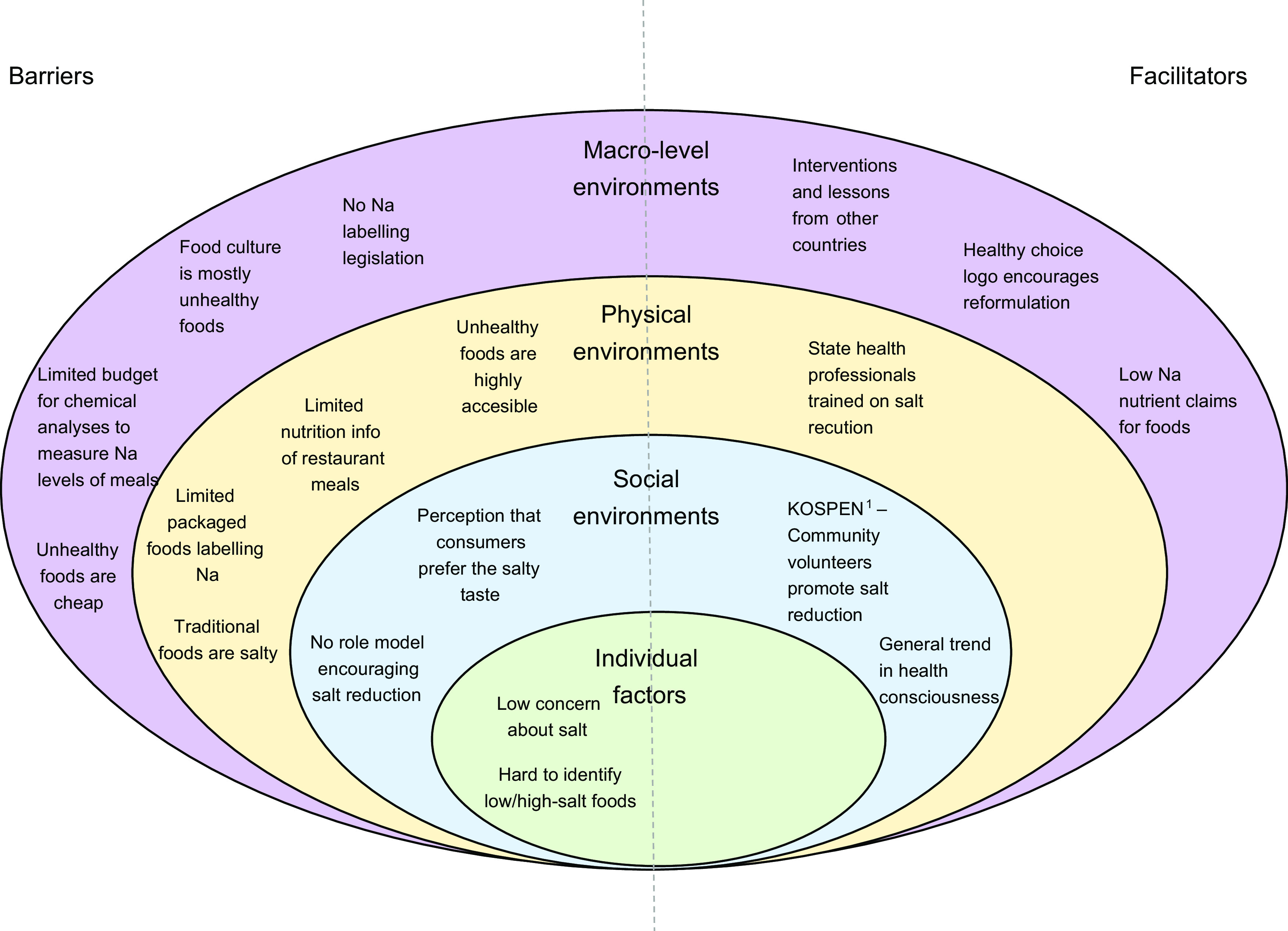
Barriers and facilitators of consuming lower salt in **Malaysia** depicted on Story et al ecological framework^(19)^. ^1^KOSPEN stands for ‘Komuniti Sihat Perkasa Negara’ translates to *Strong National Healthy Community*

**Fig. 2:**
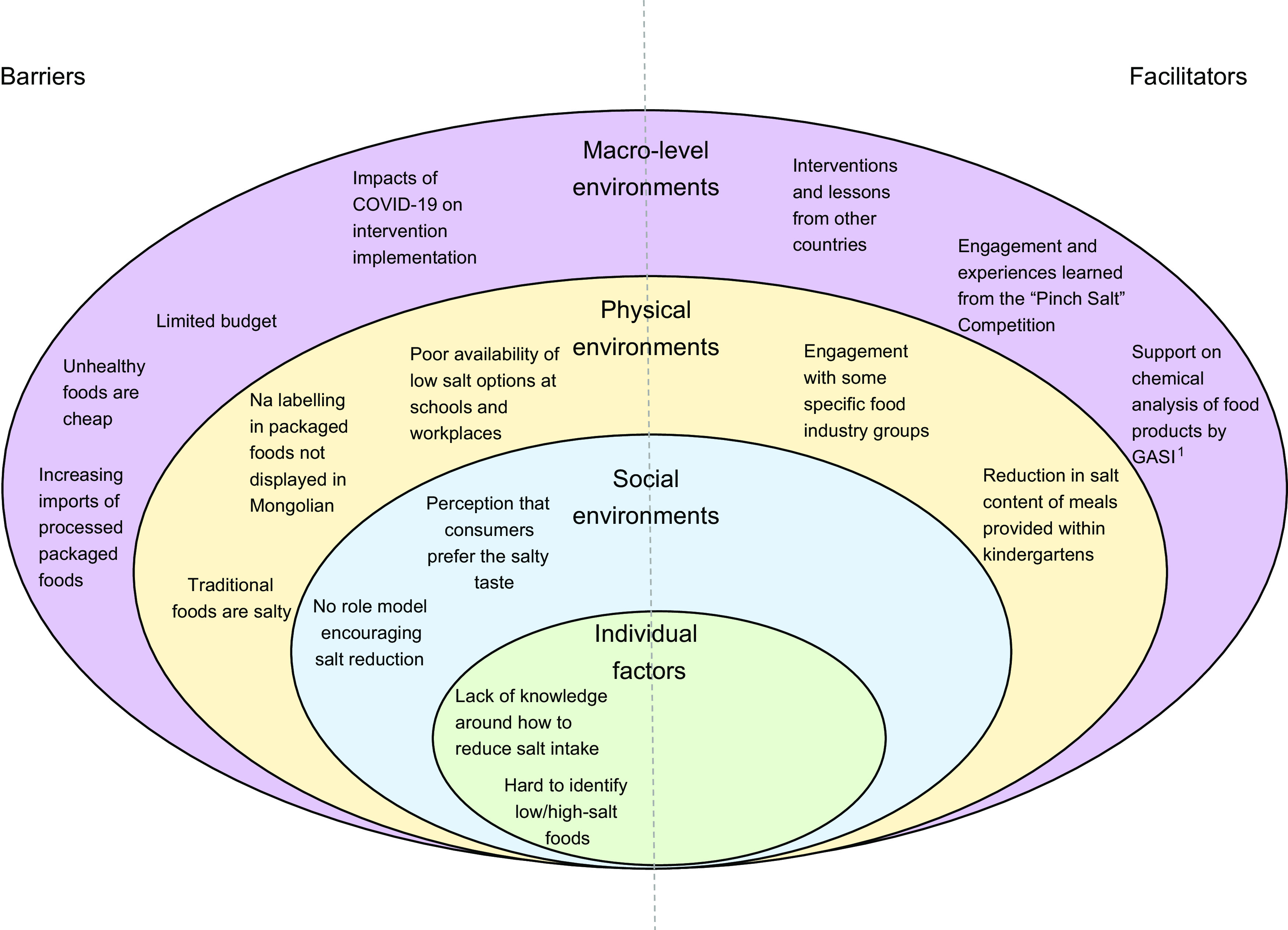
Barriers and facilitators of consuming lower salt in **Mongolia** depicted on Story et al ecological framework^(19)^. ^1^GASI stands for ‘The Generalised Agency for Specialised Inspection’

The barriers and facilitators to the implementation of salt reduction strategies, per intervention activity, are shown in Tables 3 and 4 for Malaysia and Mongolia, respectively. Barriers and facilitators to implementing the strategies overall and lowering population salt intake are shown in Figures1 and 2, depicted on Story et al’s ecological framework^(19)^. On a macro-level, barriers to implementation of both strategies included limited budget for program implementation and a perception among consumers that unhealthy (high salt) foods are more affordable than healthy (low-salt) foods. For Mongolia, the process evaluation was conducted during the initial stages of the COVID19 pandemic, which was already having an impact on strategy implementation through limitations to resources. There were also macro-level facilitators to both strategies, including the ability to learn from global best practice.

Barriers at a physical environment level for both strategies were that traditional foods in their country are salty and that unhealthy foods are highly accessible. In both countries, there were barriers with Na labelling, with limited Na labelling on packaged foods in Malaysia, and issues with Na being labelled in different languages in Mongolia. Some facilitators at a physical environment level included engagement with different actor groups, such as state health professionals in Malaysia, and specific food industry groups in Mongolia.

At a social environment level, barriers included a perception that consumers prefer salty foods and the lack of role models supporting the need for lower Na intake. In Malaysia, there was a perception that consumers are generally becoming more health conscious, which may help salt reduction efforts. However, stakeholders in both Malaysia and Mongolia identified that at an individual level, consumers found there was a lack of knowledge or concern for reducing salt and that it was difficult to identify low/high-salt food products.

## Recommendations

Based on the evaluations, and by comparing the progress to the intended aim in each strategy’s logic model (see online supplementary material, Supplementary Figures 1 and 2), two types of recommendations were generated for each country: (1) recommendations specific to the intervention components and (2) broader recommendations for the overall strategy (Table 5). For example, in Malaysia, the acceleration of the Na labelling legislation for all packaged foods was a priority recommendation because it was crucial to the implementation of potential impactful interventions (reformulation and front of pack labelling), it was highly feasible (implemented in several countries worldwide and mandated by Codex Alimentarius^(20)^), and because it addressed one of the top-ranked barriers of lowering salt intake (i.e. consumers had difficulty identifying low/high-salt food products).

**Table 5:** Recommendations derived from mid-term evaluations for salt reduction strategies in Malaysia and Mongolia

	Prioritised recommendations, based on intervention focus areas	Overall recommendations
***Malaysia***		
	Prioritise and accelerate Na labelling legislation for all packaged foods (*Products objective*)	Extend the salt reduction strategy beyond 2020
	Establish several communication channels to strategically reinforce salt reduction messages to the Malaysian population (*Awareness objective)*	Facilitate greater integration of strategies and cohesion among stakeholders leading different elements of the salt reduction strategy.
	Conduct comprehensive monitoring of the Na content of ready-to-eat processed foods or meals and out-of-home dining meals in Malaysia (*Monitoring objective*)	Conduct ongoing evaluation of process measures to inform adaptations to the interventions
***Mongolia***		
	Advocate the importance of front-of-pack labelling to food industry while exploring mandatory front-of-pack labelling (*Legal environment objective*)	Continue implementation of Mongolia’s national salt reduction strategy, with integration into the government and relevant ministries’ policies and programs
	Regularly organise a campaign or initiative to support low-salt products and production, with regular annual monitoring of salt levels in products (*Engaging private sector objective)*	Conduct a study to test the use of potassium salts in Mongolia, if financial and technical support can be identified.
	Develop a communication strategy for educating the population and decision-makers about the importance of reducing salt intake (*Supporting people to reduce salt objective)*	With the support of international and/or local organisations, determine sources of salt intake in Mongolia.

## Discussion

At the midpoint of salt reduction strategies in Malaysia and Mongolia (3 years and 5 years, respectively), both countries had undertaken high-quality salt-related surveys, developed culturally-specific education resources and trained health professionals about the importance of salt reduction. However, there were challenges in implementing legislative interventions and a comprehensive reformulation program to lower Na in foods. This study demonstrates how process evaluation methods can be applied to understand the extent as well as the barriers and facilitators of implementation and how such findings elucidate key areas for strengthening to maximise the success of strategies to lower salt intake in Malaysia and Mongolia. Given the WHO target of reducing population salt intake by 30 % by 2025 is fast approaching, process evaluations of existing salt reduction policies will be useful for strengthening their strategies.

### Policy implications

We summarise the feasibility of the WHO best buy salt reduction initiatives based on the strategies in Mongolia and Malaysia^(5)^. First, encouraging reformulation of Na levels in packaged foods on a large scale was challenging to implement when Na content labelling was not mandatory and missing on a large proportion of packaged foods in Malaysia. This is because without Na labelling, it was not possible to monitor whether products had reformulated and only engaging food companies that voluntarily labelled Na content to reformulate could discourage Na labelling. In Mongolia, there was industry opposition to reformulation. Second, we found that healthy food procurement policies were achievable in Mongolia, in line with evidence that this is the most commonly implemented salt reduction initiative worldwide^(6)^, but this was not a focus in Malaysia’s strategy. Third, both Mongolia and Malaysia developed high quality, culturally specific material for mass media and behaviour change activities; however, these were infrequently disseminated with high reach (e.g. usually once a year during World Salt Awareness Week) and often sporadic rather than being part of a strategy. One reason for this was because most modes of communication that were wide reaching (e.g. television) were costly which were identified in other studies^(10,11)^. Finally, the ability to explore or further implement front of pack labelling for Na in Mongolia and Malaysia, respectively, was limited by lack of back-of pack labelling of Na content. In Malaysia, a subsequent study identified further barriers such as lack of resources, governance complexity, industry resistance and lack of monitoring to regulations for nutrition labelling more broadly^(21)^.

Our case studies have highlighted the need for implementation of legislative policies to alter the food environment in Malaysia and Mongolia. Key recommendations from both contexts prioritised the need to accelerate legislative initiatives, as such initiatives were delayed. In Malaysia, mandatory Na labelling on all packaged foods was delayed by the process to incorporate mandatory sugar labelling. The absence of Na labelling across all packaged foods hindered the implementation of other salt reduction initiatives, which was also identified during the implementation of Samoa’s salt reduction strategy^(10)^. For example, programme implementers were unable to identify and engage food manufacturers in Na reformulation or display of the front-of-pack labelling scheme without knowledge of Na levels in packaged foods. Additionally, consumer education about reading labels and selecting lower Na products was challenging without consistent Na labelling on products, which explains why a top barrier of salt reduction in Malaysia was that it was hard for consumers to identify low- or high-salt products. While not specifically identified in the present study, there is evidence of industry interference on front-of-pack labelling in Malaysia, slowing implementation processes^(22)^. In Mongolia, legislative strategies such as mandating Na standard in foods, restricting the marketing of high-salt foods and controlling the importation of high-salt foods were not yet achieved. An effectiveness hierarchy is well established in public health nutrition literature, with the greatest health benefits coming from ‘upstream’ population-wide policy interventions changing the environment that people live within, for example by regulatory and fiscal measures, being far more effective than ‘downstream’ interventions targeting individuals, such as education and awareness raising^(23)^. In the present studies, we have highlighted those legislative initiatives need to be the focus for Malaysia and Mongolia to reach their intended outcomes.

The process evaluations also identified the need for education/behaviour change communication strategies in both countries. While both strategies had education and awareness raising campaigns, they were conducted infrequently, and therefore highlight the need to establish behaviour change strategies utilising multiple communication channels to reinforce messages and increase the message reach. These strategies could also incorporate survey findings (for example, findings on salt related knowledge, attitudes and behaviours or salt surveys on the main sources of salt) to inform the messages used. A process evaluation conducted in Fiji at the end of a national salt reduction intervention identified a similar issue, with one-off communication activities, meaning there was not sustained messaging^(11)^. A key barrier to the implementation of continual education or awareness campaigns is the identified lack of resource. However, the utilisation of different channels of communication (rather than specific mass media channels) could be a less costly method.

Our study demonstrated the methods of evaluating the implementation process of salt reduction strategies during the life of the program. Compared with previous process evaluations of salt reduction strategies that were conducted at the end of the programme to provide insights into why the programme did/did not have the expected outcomes^(10,11)^, this approach allows for changes to strengthen the strategy and ensure it is on-track to achieving the targeted salt reduction. The utility of this method is demonstrated through the adoption of almost all the recommendations in Malaysia including the mandating Na labelling on packaged foods in 2020^(24)^, continued process evaluation efforts in 2021^(25)^ and a successful grant to support the assessment of Na levels in street foods and strengthen their behaviour change communication strategy^(26,27)^. This is particularly important for salt reduction strategies that are often long-term, complex and require adaptation to the local context.

### Strengths and limitations

There are important strengths to this study. We used case studies from two different countries to illustrate how this approach can be used in different contexts and to assess the implementation of different strategies. Different data sources were used, with information triangulated to inform results and recommendations. Further, findings were discussed with in-country collaborators to check for any misinterpretations, and recommendations were presented back to strategy implementors and adapted as necessary to ensure the recommendations were feasible to adopt. Finally, lessons learned from these process evaluations of salt reduction policies are transferable to other nutrition policies (such as sugar reduction). For example, the absence of mandatory sugar labelling on packaged foods is likely to hinder the implementation of sugar reformulation policies or front-of-pack labelling schemes involving sugar.

There are also some important limitations relevant to this study. It is possible that not all available documents were assessed, or that all important people relevant to the strategies were interviewed. Further, some interview responses may be subject to optimistic bias, as stakeholders involved in implementation of the strategy were interviewed. However, this was minimised through interviewing several external stakeholders and using multiple data sources to validate the interview findings. For Mongolia, we were unable to conduct focus group discussion with health professionals, given restrictions related to the COVID19 pandemic. Instead, questionnaires were sent to health professionals. This differed from the approach taken in Malaysia and given the approach, we are unlikely to have had the same depth of information from the questionnaires as the intended group discussions. For both Malaysia and Mongolia, there were relatively small sample sizes for stakeholders interviewed and health professionals contacted. This may limit representativeness of these data.

### Conclusions

This process evaluation has demonstrated that Malaysia and Mongolia have both implemented several planned salt reduction initiatives with high fidelity, however, faced challenges in scaling-up reformulation and education initiatives to achieve high population-wide reach. Additional effort and support are needed to implement mandatory policies to encourage salt reformulation across the food supply in both countries to have population-wide impact. Other countries with salt reduction strategies should incorporate process evaluations to strengthen and accelerate their individual strategies but also generate broader lessons for countries worldwide, to achieve the WHO target of a 30 % reduction in population salt intake by 2025.

## Supporting information

McKenzie et al. supplementary material McKenzie et al. supplementary material

## Data Availability

The datasets used and/or analysed during the current study are available from the corresponding author on reasonable request and on agreement with WHO collaborators.

## Supplementary material

For supplementary material accompanying this paper visit https://doi.org/10.1017/S1368980023002781.
