# Supplementary material for: Strengthening national salt reduction strategies using multiple methods process evaluations: case studies from Malaysia and Mongolia
Source: Public Health Nutr. 2024 Feb 12;27(1):e89. doi: 10.1017/S1368980023002781 (PMC10966880; doi:10.1017/S1368980023002781)
Supplement: McKenzie et al. supplementary material [file S1368980023002781sup001.docx]

**Strengthening national salt reduction strategies using multiple methods process evaluations – case studies from Malaysia and Mongolia**

**Supplementary material**

- Supplementary Figure 1. Logic model of Malaysia’s salt reduction strategy and outputs achieved by 2018
- Supplementary Figure 2. Logic model of Mongolia’s salt reduction strategy and outputs achieved by 2020
- Supplementary information. Interview guide for semi-structured interviews with key informants in Malaysia
- Supplementary information. Interview guide for semi-structured interviews with key informants in Mongolia
- Supplementary table 3. Questionnaire on barriers and facilitators to the implementation of the national salt reduction strategy, sent to health department workers and community health workers in Mongolia

**Supplementary Figure 1.** Logic model of Malaysia’s salt reduction strategy and outputs achieved by 2018


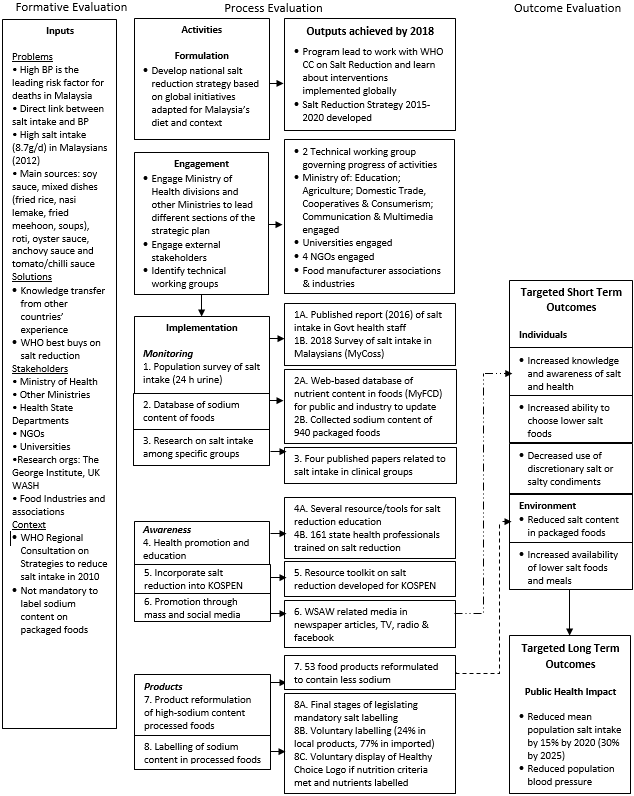


**Supplementary Figure 2**. Logic model of Mongolia’s salt reduction strategy and outputs achieved by 2020

**
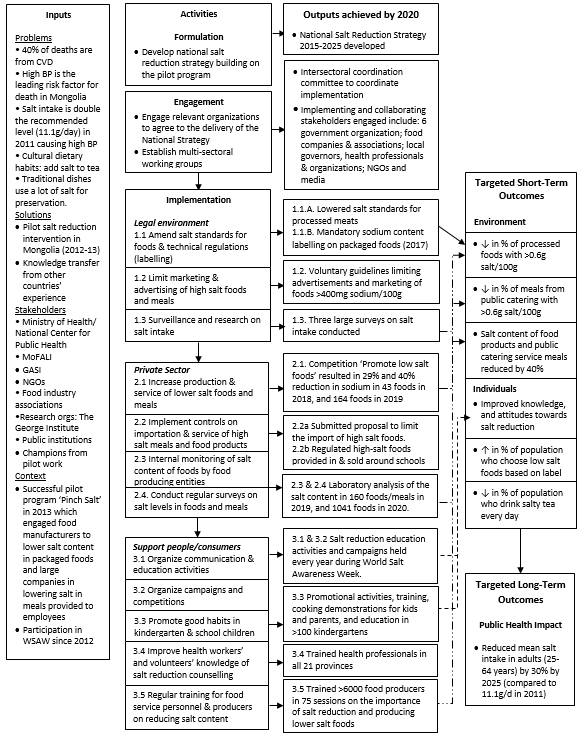
**

**Supplementary information.** Interview guide for semi-structured interviews with key informants in Malaysia

**1. Introduction and involvement**

a. Can you please introduce yourself, your position and organization for the record?

b. Can you briefly explain how you or your organization are involved in the salt reduction strategy?

c. How did your collaboration or involvement in salt reduction come about/start? (*If lead org,* How did salt reduction become a priority for your organization)

**2. Overall strategy**

a. How is the strategy governed? How well does the multi-sectoral advisory group coordinate different roles and responsibilities?

b. How is the salt reduction strategy funded? How is funding distributed through the different implementers?

c. Have you encountered any challenges in relation the overall implementation of the strategy?

d. How do you think the intervention is progressing? And Why?

e. Do you think the salt reduction strategy is on track to achieve a 30% reduction in salt intake by 2020?

**3. Monitoring** (salt intake measurement, salt levels in foods and research)

a. Are you involved in the monitoring component of the intervention? If no, how do the monitoring activities link with the initiatives that you are involved with? If yes, how do you think the monitoring activities are going compared to what was planned?

b. What challenges have you faced in regarding to implementing the monitoring initiatives?

c. What has helped facilitate the monitoring initiatives? And how?

d. How do you think the monitoring activities will help contribute to the overall goal of reduced population salt intake?

**4. Awareness** (health education and mass media)

a. Are you involved in the awareness raising initiatives of the salt reduction strategy? If no, how do the awareness raising initiatives link with the interventions that you are involved in? If yes, can you describe your approach and what activities have been implemented?

b. How is the implementation of the awareness component of the strategy going compared to what was planned? *[reach, dose, fidelity, quality]*

c. What challenges have you faced when implementing the awareness raising interventions?

d. What factors have facilitated the implementation of awareness interventions?

e. Do you think the health education and mass media initiatives will lead to improve salt-related knowledge, attitudes and behaviour in the Malaysia population, and why?

f. What has worked well and what facilitators of the intervention should be leveraged in the future?

**5. Products** (reformulation of processed food and compulsory labelling of sodium content)

a. Are you involved in the interventions related to the products (reformulation of high salt foods) and labelling of sodium content in processed foods? If no, how do you think the reformulation and labelling initiatives will contribute to the overall goal of reduced salt intake? If yes, can you describe what approaches have been taken?

b. How is the implementation of the reformulation initiatives going compared to what was planned? [adoption, food industry engagement, dose – level of sodium content reduction]

c. What challenges have you faced when trying to encourage food industry to reformulate their food products to contain less sodium?

d. What factors have driven the food industry to lower the salt content of their food products?

e. Do you think the reduced salt content of processed foods would impact the population’s salt intake? And why? [Are the reductions substantial or are they in commonly consumed foods, do you think people will consume these reduced salt foods?]

f. How is the process of mandating the labelling of sodium content on processed foods going?

g. What challenges have you faced when trying to introduce mandatory labelling of sodium content in processed food?

h. What factors have helped drive mandatory labelling of sodium content on processed foods?

g. Do you think having compulsory labelling of salt content on processed foods will influence people’s food choices, and why? Or do you think food manufacturers will make their food products healthier now that they have to label the salt content, and why?

**Other**

Was there anything else you think should be done to ensure the target of a 30% reduction in salt intake is achieved by 2020?

**Supplementary information**. Interview guide for semi-structured interviews with key informants in Mongolia

**1. Introduction and involvement**

a. Can you please introduce your position and organization?

b. Can you briefly explain your role or your organization’s role in Mongolia’s salt reduction strategy?

**2. Overall strategy**

a. Firstly, how do you think the national salt reduction strategy overall is progressing since it started in 2015?

b. There are many different organisations involved in the national strategy- how is the strategy governed?

c. What are the main challenges you have encountered in relation to implementing the overall strategy?

d. Do you think the salt reduction strategy is on track to achieve a 30% reduction in salt intake by 2025?

**3. Enabling legal environment for promoting the production, importation, marketing of low salt food (First objective)**

Now I will ask about the First objective of the salt strategy – enabling legal environment for promoting the production, importation, marketing of low salt food.

a. Are you involved in any activities related to creating an enabling legal environment for the production, importation or marketing of lower salt food (the first objective)?

If yes, can you describe your activities*?*

If no, how does the initiatives to create an enabling legal environment to promote the production, importation and marketing of low salt foods link with the interventions that you are involved in?

b. How you think these activities are going compared to what was planned?

c. What challenges have you faced when implementing these activities?

d. What has worked well or what has helped with the implementation of these activities?

e. Do you think these activities related to creating an enabling legal environment for promoting the production, importation and marketing of low salt foods will lead to the goal of lowering salt intake in Mongolia?

**4. Engaging the private sector to reduce salt content of foods (second objective)**

Now I will ask about the second objective of the salt strategy – engaging the private sector to reduce salt content in foods, and increasing controls on the production, service, marketing, importation and consumption of food.

a. Are you involved in the initiatives to engage the private sector to reduce the salt content of foods?

If yes, can you describe your activities*?*

If no, how does the initiatives to engage the private sector in salt reduction link with the interventions that you are involved in?

b. How you think these activities are going compared to what was planned?

c. What challenges have you faced when implementing these activities?

d. What has worked well and what factors have made the implementation of these activities easier?

e. Do you think engaging the private sector to reduce the salt content in foods will lead to lower salt intake in Mongolia?

**5. Creating an enabling environment to support people/consumers lower salt intake**

Now I will ask about the third objective of the salt strategy – creating an enabling environment which supports people to develop habits of adequate use of salt and make the right choices of meals and food products.

a. Are you involved in the initiatives to create an environment to support people and consumers to lower salt intake?

If yes, can you describe your activities?

If no, how does the initiatives to create an environment to support people to lower salt intake link with the interventions that you are involved in?

b. How you think these activities are going compared to what was planned?

c. What challenges have you faced when implementing these activities?

d. What has worked well and what factors have made the implementation of these activities easier?

e. Do you think creating an enabling environment to support consumers to lower salt intake will lead to lower salt intake in Mongolia?

**Other**

Was there anything else you think should be done to help lower salt intake in Mongolia?

**Supplementary table 1.** Questionnaire on barriers and facilitators to the implementation of the national salt reduction strategy, sent to health department workers and community health workers in Mongolia

| Question | 1. Response | Rank |
| --- | --- | --- |
| What factors (barriers) do you think make it hard for individuals in Mongolia to reduce their salt intake? | | |
| Lack of concern in regard to salt consumption | Agree or disagree?  Comments: |  |
| Lack of knowledge of the adverse health effects of excess salt consumption | Agree or disagree?  Comments: |  |
| Lack of knowledge of how to reduce salt intake | Agree or disagree?  Comments: |  |
| Taste preference for salty foods and drinks | Agree or disagree?  Comments: |  |
| Please list other factors that you think make it hard for people in Mongolia to reduce their salt intake? | | |
| Factor: | Comment: |  |
| Factor: | Comment: |  |
| What social factors (e.g. social norms, or social support from friends and family) do you think make it hard for communities in Mongolia to reduce their salt intake? | | |
| Eating out is an increasing social trend and food from restaurants, fast food or takeaway is unhealthy and high in salt | Agree or disagree?  Comments: |  |
| A lack of role models who encourage reducing salt intake | Agree or disagree?  Comments: |  |
| Other social factors (e.g. social norms, or social support from friends and family) that you think make it hard for communities in Mongolia to reduce their salt intake? | | |
| Factor: | Comment: |  |
| Factor: | Comment: |  |
| What physical environmental factors (e.g. workplace, schools, restaurants, supermarkets, convenience stores) do you think make it hard for individuals/communities in Mongolia to reduce their salt intake? | | |
| Limited access to healthy, low-salt food options in schools, workplace or hospital canteens | Agree or disagree?  Comments: |  |
| Unhealthy, high-salt processed and packaged foods are easily accessible compared to healthy, low-salt foods (fruit and vegetables) | Agree or disagree?  Comments: |  |
| Other physical environmental factors (e.g. workplace, schools, restaurants, supermarkets, convenience stores) that you think make it hard for individuals/communities in Mongolia to reduce their salt intake? | | |
| Factor: | Comment: |  |
| Factor: | Comment: |  |
| What macro-level (e.g. governmental policies, food industry, food production and agriculture policies) factors make it hard for individuals/communities in Mongolia to reduce their salt intake? | | |
| Cost of unhealthy, high salt foods are cheaper than healthy, low salt foods (e.g. fruit and vegetables) | Agree or disagree?  Comments: |  |
| Traditional foods are mostly high in salt | Agree or disagree?  Comments: |  |
| Other macro-level factors that you think make it hard for individuals/communities in Mongolia to reduce their salt intake? | | |
| Factor: | Comment: |  |
| Factor: | Comment: |  |
| *3. Now please review your response and then rank the importance of these factors, with “1” being the LEAST important barrier to salt reduction in Mongolia, in ascending order to the MOST important factor being the largest number for example “18”* | | |
